# Using Administrative Data to Assess the Risk of Permanent Work Disability: A Cohort Study

**DOI:** 10.1007/s10926-020-09926-7

**Published:** 2020-09-10

**Authors:** Matthias Bethge, Katja Spanier, Marco Streibelt

**Affiliations:** 1 Institute for Social Medicine and Epidemiology, University of Lübeck, Ratzeburger Allee 160, 23562 Lübeck, Germany; 2 Federal German Pension Insurance, Berlin, Germany

**Keywords:** Pensions, Rehabilitation, Needs assessment, Employment, Social welfare, Longitudinal studies

## Abstract

*Purpose* Unmet rehabilitation needs are common. We therefore developed a risk score using administrative data to assess the risk of permanent work disability. Such a score may support the identification of individuals with a high likelihood of receiving a disability pension. *Methods* Our sample was a random and stratified 1% sample of individuals aged 18–65 years paying pension contributions. From administrative records, we extracted sociodemographic data and data about employment and welfare benefits covering 2010–2012. Our outcome was a pension due to work disability that was requested between January 2013 and December 2017. We developed a comprehensive logistic regression model and used the model estimates to determine the risk score. *Results* We included 352,140 individuals and counted 6,360 (1.8%) disability pensions during the 5-year follow-up. The area under the receiver operating curve was 0.839 (95% CI 0.834 to 0.844) for the continuous risk score. Using a threshold of ≥ 50 points (20.2% of all individuals), we correctly classified 80.6% of all individuals (sensitivity: 71.5%; specificity: 80.8%). Using ≥ 60 points (9.9% of all individuals), we correctly classified 90.3% (sensitivity: 54.9%; specificity: 91.0%). Individuals with 50 to < 60 points had a five times higher risk of a disability pension compared to individuals with low scores, individuals with ≥ 60 points a 17 times higher risk. *Conclusions* The risk score offers an opportunity to screen for people with a high risk of permanent work disability.

**Electronic supplementary material:**

The online version of this article (10.1007/s10926-020-09926-7) contains supplementary material, which is available to authorized users.

## Introduction

Population ageing and the transition from communicable to non-communicable diseases increase the number of people living with disabilities [1]. There are globally more than 1 billion people with disabilities; by 2050, the World Health Organization expects that the number will rise to over 2 billion [2]. Their equal participation is one of the major challenges that our health and social security systems are facing [3, 4].

Paid employment is one of the life domains in which there is a particular need for action. Employment secures income, guarantees material security, and supports an independent lifestyle. In addition, the accumulation of pension entitlements reduces the risk of poverty in old age. However, employment rates among people with disabilities are only half those of people without disabilities in many countries [5]. Enhancing the work participation of people with chronic and severe health impairments requires effective rehabilitation strategies that reduce the consequences of illness and find ways to enable people with health impairments to return to work. In many European countries, pension and health insurance agencies provide rehabilitation programs for this purpose [6]. These rehabilitation programs aim to prevent permanent work disability and the need for disability pensions by restoring and improving work ability. Although these services are available, and the possibility of utilization is—as in Germany—guaranteed by law, unmet needs are common. In Germany, there are about 170,000 new disability pensioners each year, and roughly half of them did not use rehabilitation before receiving a disability pension [7]. In order to ensure access to rehabilitation for all who need it, persons at increased risk of permanent work disability have to be identified in time.

The increasing accessibility of digital health and employment data has renewed interest in the use of these data for research and developing health care [8]. These data are easily available and can be processed in real time. If these data can be effectively used to identify people at high risk of permanent work disability, this would open up new avenues for targeted information on rehabilitation services. In Germany, the pension agencies routinely collect data on employment and welfare benefits, in particular to determine later pension entitlements, but they are also responsible for providing rehabilitation for working-aged people. Many of the data routinely collected are known to be associated with future disability pensions. Based on a previous case–control study, we therefore proposed to merge these data, assuming that this would offer more insight into the risk of permanent work disability than looking at the various distinct pieces of data separately [9]. In our present study, we sought to weight and combine various administrative sociodemographic data and data on employment and welfare benefits into a single score which accurately predicts future disability pensions. Moreover, we intended to determine a reasonable threshold that separates individuals with low and high risks of permanent work disability.

## Methods

### Study Design and Participants

Our study is a cohort study employing administrative data which are routinely collected by pension agencies. We used a random 1% sample of individuals aged 18 to 65 years who were paying pension contributions in 2012, stratified by pension insurance institutions. Excluded were persons who applied for an old age or disability pension before 1 January 2013. The study protocol was approved by the ethics committee of the University of Lübeck (18-246A).

### Outcome

Our outcome was a disability pension claimed between 1 January 2013 and 31 December 2017. In Germany, there is a compulsory pension insurance scheme. In total, there are 16 agencies which currently administer the pension contributions of around 55 million working-aged people, of whom about 38 million had paid contributions in 2017. In the case of lasting work disability, the agencies have to pay a disability pension. Disability pensions can be approved as full or partial pensions: about 90% are full pensions [7]. Disability pensions are usually permitted temporarily (up to 3 years), and a continuation of the pension needs further verification. Once approved, a later refusal is rather rare. Temporary pensions turn into permanent pensions after 9 years. Data on disability pensions were extracted from administrative records. Since temporary pensions usually turn into permanent pensions we did not distinguish between temporary and permanent pensions.

### Independent Variables

Administrative sociodemographic data and data on employment and welfare benefits were used to predict disability pensions. The sociodemographic characteristics taken into account were age, gender, nationality, and the pension agency. Nationality was categorized as German, Turkish, former Yugoslavia, Russian and Commonwealth of Independent States, Polish, Italian, Greek, and other. Persons with German citizenship formed the reference category in our model calculations. The reference category for the pension agency was the Federal German Pension Insurance, which is the largest agency mainly covering white-collar workers. Employment was represented by cumulative income (in 1000 euros) for the years 2010 to 2012. The duration of welfare benefits (short-term unemployment benefit, long-term unemployment benefit, and sickness absence benefit) was also cumulated for 2010 to 2012 and then categorized. For this purpose, the median was determined for persons who were entitled to welfare benefits for at least 1 day, and the duration of benefits was then categorized as no benefits vs. short benefits vs. long benefits.

### Statistical Analysis

Sample characteristics were determined using descriptive methods. Subsequently, disability pensions were regressed on all independent variables using simple logistic regression models. For each characteristic, both the crude association and interactions with gender were tested. If we identified a significant interaction effect, sex-specific estimators were considered in the final model. In the final model, we included sex, age, nationality, the pension agency, income, the duration of short-term and long-term unemployment benefits, and the duration of sickness absence benefits. In the case of income, the duration of long-term unemployment benefits, and the duration of sickness absence benefits, sex-specific variables were included to account for the significant interaction in the crude models. In order to consider the non-linear effect of age in predicting a disability pension, the squared age was also included. Unstandardized estimates and odds ratios with corresponding 95% confidence intervals were determined as estimators. To calculate our risk score, the unstandardized estimators and the characteristics of the included individuals were combined linearly and then transformed into probabilities before being converted into T-scores with a mean of 50 and a standard deviation of 10.

The prognostic accuracy of the continuous risk score was assessed using a receiver operating characteristic curve and the corresponding area under the curve. A value of > 0.5 implies that the predictive power is better than random; values of ≥ 0.8 are considered good [10]. As the apparent performance of a prognostic model on a development sample is usually better than the performance on other samples, even if the latter sample consist of individuals from the same population we internally validated our model [11]. We used the bootstrapping technique described by Harrell et al. [12] with 200 repetitions. To assess the prognostic accuracy of a categorized risk score, we calculated sensitivity and specificity, the correct classification rate, and the positive and negative likelihood ratios for each potential threshold. The index J (J = sensitivity + specificity − 1) proposed by Youden was used to determine the optimal threshold for categorization of our risk score [13]. The optimized threshold was determined as the score for which the Youden index reached its maximum. Finally, Kaplan–Meier curves were generated to describe the prognostic relevance of our categorized risk score. Time-to-event was computed from 1 January 2013 until the date of application for an approved disability pension. The serial time of people who received an old-age pension was censored at the date of start of the old-age pension. Individuals’ serial time without an event ended on 31 December 2017. In addition, a proportional hazard model using the categorized risk score as the independent variable was calculated, and hazard ratios and their 95% confidence intervals were determined.

Statistical tests were considered significant if the two-tailed level of significance was less than 5%. All analyses were calculated using STATA version 15.

## Results

### Sample Characteristics

We included 352,140 individuals and counted 6360 (1.8%) disability pensions during the 5-year follow-up. Table 1 shows the sample characteristics for individuals with and without a disability pension separately. Persons who received a disability pension during the 5-year follow-up were older, were less often customers of the Federal German Pension Insurance, were more often Turkish nationals, achieved less income, and received welfare benefits for longer.

**Table 1:** Sample characteristics

	Received disability pension			No disability pension		
	N	%	Mean (SD)	N	%	Mean (SD)
Sex						
Male	3225	50.7		178,033	51.5	
Female	3135	49.3		167,747	48.5	
Age in years	6360		49.5 (8.4)	345,780		41.3 (12.3)
Pension insurance agency^a^						
Federal German Pension Insurance	2131	33.5		155,008	44.8	
Other	4229	66.5		190,772	55.2	
Nationality						
German	5696	89.6		309,195	89.4	
Turkish	248	3.9		8,789	2.5	
Former Yugoslavia	78	1.2		3,818	1.1	
Russian and Commonwealth of Independent States	45	0.7		2,597	0.8	
Polish	29	0.5		2,554	0.7	
Italian	50	0.8		2,519	0.7	
Greek	24	0.4		1,292	0.4	
Other	190	3.0		15,016	4.3	
Income in 1000 euros^b^	6360		47.0 (45.2)	345,780		64.4 (58.0)
Duration of short-term unemployment benefits in days^b^	6360		41.7 (103.2)	345,780		19.5 (68.9)
Duration of short-term unemployment benefits^b^						
None	5066	69.7		303,882	87.9	
Short (1 to 130 days)	460	8.7		21,138	6.1	
Long (> 130 days)	834	21.6		20,760	6.0	
Duration of long-term unemployment benefits in days^b^	6360		194.5 (328.1)	345,780		61.1 (198.0)
Duration of long-term unemployment benefits^b^						
None	4435	69.7		305,455	88.3	
Short (1 to 624 days)	554	8.7		20,575	6.0	
Long (> 624 days)	1371	21.6		19,750	5.7	
Duration of sickness absence benefits in days^b^	6360		103.8 (165.1)	345,780		12.0 (53.4)
Duration of sickness absence benefits^b^						
None	3423	53.8		292,272	84.5	
Short (1 to 29 days)	444	7.0		27,809	8.0	
Long (> 29 days)	2493	39.2		25,699	7.4	

n = 352,140

*SD* standard deviation

^a^The category ‘other’ insurance institutions includes 15 institutions. In our logistic regression model, these agencies were considered as separate dummy variables

^b^Data were cumulated for the years 2010 to 2012

### Determinants of a Disability Pension

The final model is available as Supplementary File 1. Men had slightly higher odds of a disability pension compared to women in this model. Higher age and lower income were associated with an elevated risk of a disability pension. The odds of a disability pension were slightly higher for Turkish people compared to those of German nationality. Persons of Russian nationality and those from the Commonwealth of Independent States, Poland, and people of other nationalities had lower odds of a disability pension compared to Germans. Compared to the Federal German Pension Insurance, the odds of a disability pension were slightly higher for almost all other pension insurance institutions. The length of time in receipt of short-term or long-term unemployment benefits, as well as sickness absence benefits, clearly increased the risk of a disability pension. In particular, prolonged receipt of long-term unemployment benefits and sickness absence benefits raised the likelihood of a disability pension.

### Risk Score

**Fig. 1:**
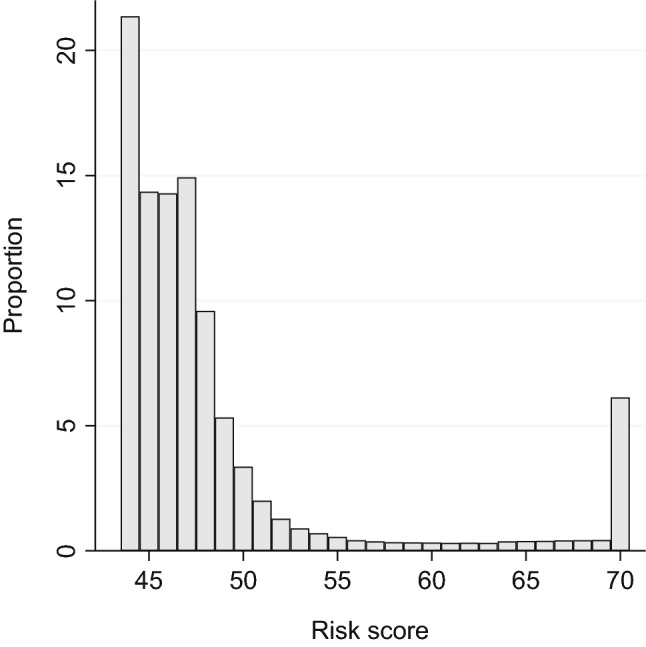
Distribution of the risk score. *Note* n = 352,140. The bars represent the integer part of the T-score. Values ≥ 70 points were combined in one category

The distribution of the risk score was right-skewed, with the mass of the distribution on the left and a long tail on the right. About one quarter had scores below 45 points. The median was 47 points. About one in five people had a score of at least 50 points, and about every tenth person had a score of at least 60 points (Fig. 1).

The risk score clearly discriminated individuals with and without disability a pension. The area under the receiver operating curve was 0.839 (95% CI 0.834 to 0.844). Internal validation showed that optimism tended to be zero (< 0.001), meaning that our apparent area under the curve was not overoptimistic. The optimal threshold determined by maximizing the Youden index was 50 points (Table 2). Using the threshold of ≥ 50 points, we correctly classified 80.6% of all individuals (sensitivity: 71.5%; specificity: 80.8%). Using ≥ 60 points, we correctly classified 90.3% (sensitivity: 54.9%; specificity: 91.0%).

**Table 2:** Prognostic accuracy of the risk score

Threshold	Pr (%)	Se (%)	Sp (%)	CCR (%)	LR +	LR-	J
≥ 45	78.6	99.2	21.7	23.1	1.27	0.04	0.21
≥ 50	20.2	71.5	80.8	80.6	3.72	0.35	0.52
≥ 55	11.9	60.2	89.0	88.5	5.47	0.45	0.49
≥ 60	9.9	54.9	91.0	90.3	6.08	0.50	0.46

n = 352,140

*Pr* prevalence, *Se* sensitivity, *Sp* specificity, *CCR* correct classification rate, *LR* likelihood ratio, *J* Youden's J statistic

**Fig. 2:**
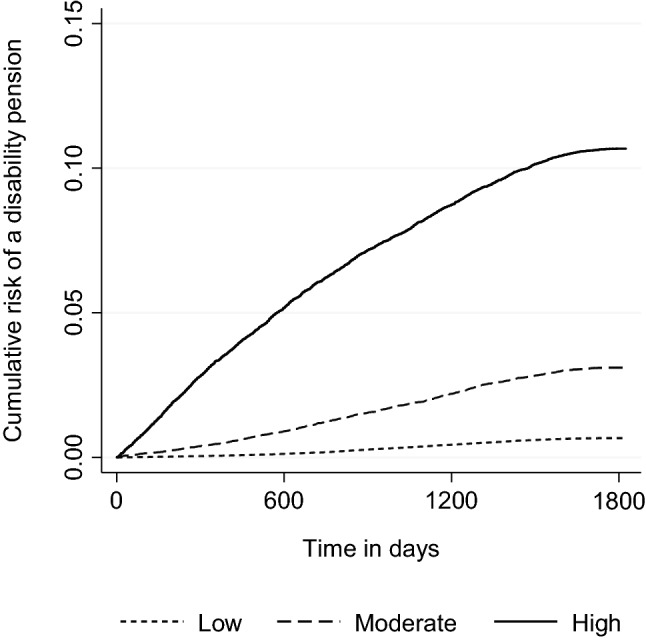
Cumulated risk of a disability pension between 2013 and 2017. *Note* n = 352,140; 6,360 disability pensions

The probability of a disability pension for persons with values < 50 points was 0.7%; for persons with values 50 to < 60 points, it was 2.9%; and for persons with values ≥ 60 points, it was 10.1% (Table 3). Figure 2 shows the cumulative risk of the three risk groups over the 5-year follow-up period. Individuals with moderate (50 to < 60 points) or high risk scores (≥ 60 points) had a 5 times (HR = 4.71; 95% CI 4.24 to 4.94) or 17 times higher risk (HR = 17.32; 95% CI 16.37 to 18.34) of a disability pension compared to individuals with low scores.

**Table 3:** Probability of a disability pension for the categorized risk score

Risk score	Received disability pension	No disability pension	Total	PPV (%)
Low	1813	279,357	281,170	0.7
Moderate	1053	35,193	36,246	2.9
High	3494	31,230	34,724	10.1
Total	6360	345,780	352,140	1.8

*PPV* positive predictive value; low: < 50 points; moderate: 50 to < 60 points; high: ≥ 60 points

## Discussion

The aim of the study was to predict the risk of a disability pension using administrative data from the German Pension Insurance and to develop a risk score, which can be used to identify unmet rehabilitation needs. The prognostic accuracy of the risk score was acceptable and similar to that of well-established risk scores in health care settings like the management of cardiovascular risk [14]. Internal validation of the model performance using bootstrapping revealed that the model was not overoptimistic. The hazards of receiving a disability pension were 5 and 17 times higher in people with moderate and high risk scores than in people with low risk scores. The number of false positive results was, however, high. Due to the low incidence of disability pensions, most people with high risk scores were still employed at the end of our 5-year follow-up.

The most important predictor of a disability pension was the duration of sickness absence benefits. These results are consistent with the findings of other large cohort studies which also examined the effect of long-term sickness absence benefits on disability pensions [15–21], and findings from an earlier case–control study in which we initially tested our idea of a risk score based on administrative data [9]. Though many studies have identified relevant risk factors for disability pensions, they did not combine these data into a single risk score. However, we believe that the overall burden of risk factors should be taken into account when assessing the individual risk of a permanent work exit. Many people who later receive a disability pension are affected by several risk factors. These factors interact with each other and thus generate the overall individual risk.

The use of administrative data to identify persons who are likely to leave the labor force due to health reasons and receive a disability pension is an appealing idea. A screening of administrative data harnesses complete data, avoids recall bias and can be applied rapidly, with little effort and low costs even for large groups. This could help to reduce the high number of disability pensions if early detection and intervention are effective. However, the requirements for an effective screening program go beyond acceptable sensitivity and specificity. Wilson and Jungner [22], in their seminal paper on the principles and practice of screening, formulated essential requirements for the implementation of screening half a century ago. Some of these requirements are certainly met. Permanent work incapacity is an important individual and social problem, and the number of disability pensions is sufficiently high to warrant preventive strategies. There is also evidence that effective strategies are feasible to support the work participation of people with chronic health problems [23–27]. Other challenges could be solved in principle, but solutions are not yet well established. Firstly, acceptance of the collection and processing of personal data by state institutions is low, and health-related data are particularly worthy of protection [28]. If they become freely available, assumptions about future restrictions could have significant negative consequences for the individuals in question and, for instance, reduce promotion opportunities or the likelihood that temporary jobs become permanent jobs. However, resistance to institutional data collection and data analysis is significantly lower when people expect a health benefit from it [28]. This benefit must be communicated in a comprehensible way. Secondly, facilities must be available to meet the increased care needs that arise for definitive diagnostic clarification and any necessary rehabilitative interventions. Thirdly, full diagnosis and treatment of people with high risk scores would cause considerable costs, although the initial costs for screening administrative data are negligible. A work disability screening using administrative data may not be cost-effective, even if a randomized controlled trial may prove that work retention is improved in comparison to a non-screened population.

A critical appraisal of our findings has to consider the following limitations. Firstly, the data we used are collected primarily for the calculation of future pensions. Thus, the purpose of the data is not to assess rehabilitation needs. Important information, such as the type of illness, is not available, as this information is not relevant for the calculation of the pension amount. This limits the sensitivity and specificity of our risk score. Secondly, a critical editorial pointed out that performance of recently developed prediction models is not good enough for implementing risk-based intervention strategies [29]. While these models demonstrated the importance of certain risk factors they were mostly not sufficient to accurately identify individuals at risk of disability benefits and to target these individuals for interventions that permanently reduce this risk. In line with these findings, our low positive predictive value indicates that our risk score is—at best—suitable for screening but not appropriate to directly assign rehabilitation services. Thirdly, though we internally validated our model using bootstrapping we have not externally validated our model. While internal validation did not reveal optimism the model performance in independently established cohorts may be worse [30–32]. Fourthly, the employment and income data used are not reported in real time by employers, but only after the end of the year. The risk score may refer to an event that has already been overcome. Interventions may have been initiated, or the favorable course of an illness may have already resolved the limitations of the persons concerned.

These limitations are countered by the following strengths. Firstly, we were able to use a large random sample for the development of our risk score. The external validity of our results is therefore high. Secondly, the administrative data we used as independent variables and outcome were complete, reliable, and valid. Thirdly, we used 3-year cumulated data to predict disability pensions rather than single measurements only.

In conclusion, our risk score can be calculated for every person aged 18 to 65 years paying pension contributions in Germany. If the necessary data are available in time, this yields two key applications. Firstly, the risk score can be used as an additional evaluation criterion when assessing rehabilitation and pension requests. Secondly, there is the possibility of identifying people with high risk scores and informing them about rehabilitation services. Subsequently, it is possible to discuss whether rehabilitative services can enable them to remain in working life.

## Electronic supplementary material

Below is the link to the electronic supplementary material.

Supplementary file1 (DOCX 16 kb)
