# Supplementary material for: Using Administrative Data to Assess the Risk of Permanent Work Disability: A Cohort Study
Source: J Occup Rehabil. 2020 Sep 10;31(2):376–82. doi: 10.1007/s10926-020-09926-7 (PMC8172482; doi:10.1007/s10926-020-09926-7)
Supplement: Supplementary file 1 — Supplementary file1 (DOCX 16 kb) [file 10926_2020_9926_MOESM1_ESM.docx]

Supplementary File 1: Final logistic regression model to predict a disability pension between 2013 and 2017

|  | OR | 95% CI | p |
| --- | --- | --- | --- |
| Sex: male | 1.517 | 1.341; 1.715 | <0.001 |
| Age in years | 1.448 | 1.411; 1.486 | <0.001 |
| Age in years (squared) | 0.997 | 0.996; 0.997 | <0.001 |
| Nationality |  |  |  |
| *Turkish* | 1.251 | 1.091; 1.435 | <0.001 |
| *Former Yugoslavia* | 0.862 | 0.682; 1.090 | 0.215 |
| *Russian and Commonwealth of Independent States* | 0.660 | 0.488; 0.894 | 0.007 |
| *Polish* | 0.624 | 0.429; 0.909 | 0.014 |
| *Italian* | 0.863 | 0.644; 1.158 | 0.326 |
| *Greek* | 0.885 | 0.584; 1.340 | 0.563 |
| *Other* | 0.650 | 0.559; 0.754 | <0.001 |
| Pension insurance agency |  |  |  |
| *North* | 1.219 | 1.075; 1.382 | 0.002 |
| *Central Germany* | 0.848 | 0.757; 0.950 | 0.004 |
| *Brunswick-Hanover* | 1.303 | 1.151; 1.474 | <0.001 |
| *Westphalia* | 1.263 | 1.126; 1.417 | <0.001 |
| *Hesse* | 1.739 | 1.530; 1.976 | <0.001 |
| *Rhineland* | 1.171 | 1.044; 1.312 | 0.007 |
| *Southern Bavaria* | 1.155 | 1.001; 1.331 | 0.048 |
| *Rhineland-Palatinate* | 1.629 | 1.409; 1.883 | <0.001 |
| *Saarland* | 1.842 | 1.435; 2.364 | <0.001 |
| *Northern Bavaria* | 1.454 | 1.261; 1.677 | <0.001 |
| *Swabia* | 1.193 | 0.934; 1.523 | 0.158 |
| *Baden-Wuerttemberg* | 1.292 | 1.155; 1.444 | <0.001 |
| *Berlin-Brandenburg* | 1.115 | 0.983; 1.264 | 0.091 |
| *Oldenburg-Bremen* | 1.132 | 0.898; 1.426 | 0.293 |
| *Knappschaft-Bahn-See* | 1.632 | 1.455; 1.830 | <0.001 |
| Income in 1000 euros |  |  |  |
| *Man* | 0.993 | 0.993; 0.994 | <0.001 |
| *Woman* | 0.997 | 0.996; 0.998 | <0.001 |
| Duration of short-term unemployment benefits*^‡^ |  |  |  |
| *Short (1 to 130 days)* | 1.293 | 1.167; 1.432 | <0.001 |
| *Long (>130 days)* | 1.501 | 1.380; 1.632 | <0.001 |
| Duration of long-term unemployment benefits in days*^§^ |  |  |  |
| *Man and short (1 to 624 days)* | 1.617 | 1.413; 1.851 | <0.001 |
| *Man and long (>624 days)* | 4.740 | 4.207; 5.341 | <0.001 |
| *Woman and short (1 to 624 days)* | 1.723 | 1.483; 2.002 | <0.001 |
| *Woman and long (>624 days)* | 6.355 | 5.670; 7.123 | <0.001 |
| Duration of sickness absence benefits*^§^ |  |  |  |
| *Man and short (1 to 29 days)* | 1.901 | 1.645; 2.198 | <0.001 |
| *Man and long (>29 days)* | 6.701 | 6.161; 7.288 | <0.001 |
| *Woman and short (1 to 29 days)* | 1.881 | 1.626; 2.176 | <0.001 |
| *Woman and long (>29 days)* | 10.868 | 9.990; 11.824 | <0.001 |

Note: n = 352,140; OR = odds ratio; CI =confidence interval. * Data were cumulated for the years 2010 to 2012. ^‡^ Reference was no short-term unemployment benefits. ^§^ The reference was the sex-specific ‘none’ category.
